# Use of a Refresher Course Increases Confidence in Point-of-Care Ultrasound Skills in Emergency Medicine Faculty

**DOI:** 10.7759/cureus.5413

**Published:** 2019-08-17

**Authors:** Madeline Schwid, Owen Harris, Adaira Landry, Andrew Eyre, Patricia Henwood, Heidi Kimberly

**Affiliations:** 1 Emergency Medicine, Brigham & Women’s Hospital, Harvard Medical School, Boston, USA; 2 Emergency Medicine, Brigham and Women's Hospital, Harvard Medical School, Boston, USA

**Keywords:** emergency medicine, emergency ultrasound, medical education, faculty education, curriculum development, point of care ultrasound, skills training

## Abstract

Introduction

All practicing emergency medicine (EM) physicians need to maintain a skillset in emergency ultrasound (US) after their initial training. EM physicians in academic practice may be supervising trainees performing ultrasound applications that they aren’t comfortable with. This study investigates the effectiveness of a US refresher course. The hypothesis was that a series of short courses would increase confidence in performing and supervising US applications.

Methods

Nine basic emergency ultrasound applications were taught over the course of one year by ultrasound fellowship-trained EM faculty in a simulation center at a single academic institution. Each session included 30-minutes of didactics/image review and 30-minutes of hands-on practice on normal volunteers and was followed by an anonymous questionnaire evaluating comfort level performing and supervising the ultrasound application before and after the course using a Likert scale from 1 “not at all confident” to 5 “very confident”.

Results

Thirty-six of 60 EM physicians participated in at least 1 of the 9 sessions (median 3, interquartile range 2-4). Faculty who attended had a median of 10 (interquartile range 7-15) years in practice and 61% work at both academic and community sites.

For all sessions combined, confidence in performing US increased from a mean score on the Likert scale of 3.3 to 4.4 (difference 1.1, confidence interval (CI) (0.94, 1.29), p < 0.001) and confidence in supervising trainees increased from a mean of 3.4 to 4.5 (difference 1.1, CI (0.88, 1.23), p < 0.001). The largest increases were seen in musculoskeletal (MSK), nerve, and pelvic applications and the least increase was seen with the session focused on intravenous access, but confidence was increased in all sessions.

Physicians in practice ≥10 years increased in confidence in performing and supervising the applications by 1.4 (CI (1.11, 1.60), p < 0.001) and 1.3, (CI (1.01, 1.49), p < 0.001), respectively. Physicians in practice <10 years increased 0.8 (CI (0.57, 1.03), p < 0.001) and 0.8 (CI (0.55, 1.05), p < 0.001), respectively.

Conclusion

An emergency ultrasound refresher course for EM physicians at a single institution improved self-reported confidence in both performing and supervising trainees in all applications reviewed. Those in practice ≥10 years showed the largest increases.

## Introduction

Point-of-care ultrasound (POCUS) has become an essential tool in emergency medicine (EM) [1]. Ultrasound (US) is user-dependent and requires targeted training to ensure and maintain competence [2]. POCUS training during EM residency has recently been integrated into the Accreditation Council for Graduate Medical Education (ACGME) requirements for graduation [3]. Pathways for credentialing during residency and as a practicing EM physician have been established by the American College of Emergency Physicians (ACEP). Per policy, ongoing education after credentialing is also required for practicing physicians [4], but the exact nature of this education is left to the discretion of individual departments [5]. Low confidence in supervising physicians can be a potential source of suboptimal outcomes in the department and residency training [6-7]. Focused education beyond just clinical practice is necessary for effective learning, teaching, and patient safety [8].

Current literature has investigated different teaching models for introducing POCUS competency at various levels of training [9-12]. At the attending level, there has been a little investigation into the ideal method of maintaining US skills and comfort with the supervision of trainees. Despite efforts to standardize POCUS training during EM residency, a large variety of skills and comfort level persists after graduation [13-15].

The purpose of this study is to investigate a longitudinal US refresher course utilizing a blended model of didactic and image review with hands-on practice as a potential way for practicing EM faculty improve confidence. The hypothesis is that a series of short courses would increase confidence in performing POCUS applications in attending EM physicians. Changes in confidence for supervising trainees and the differences observed in participants based on the number of years in practice was also investigated.

## Materials and methods

**Figure 1:**
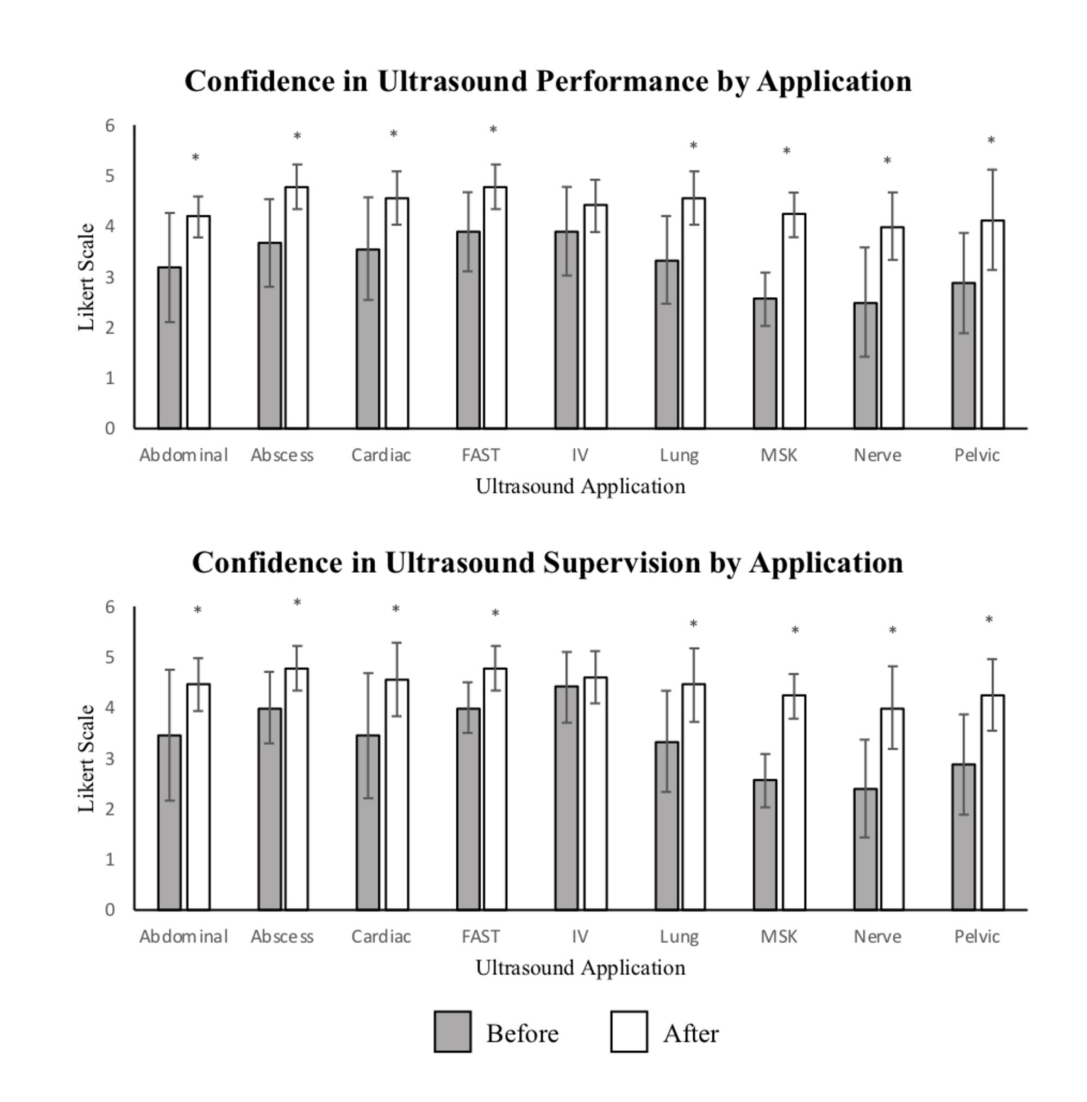
Confidence in Ultrasound Performance and Supervision by Application Confidence in US performance and supervision before and after completion of course by the specific application using the Likert scale (1-5). Statistically significant difference (p < 0.05) between responses before and after session is demonstrated by (*). Abbreviations: FAST= focused assessment with sonography in trauma, IV= intravenous, MSK= musculoskeletal

This study was performed at a single university-affiliated ED with an active residency program that is responsible for staffing a level 1 tertiary care ED, community ED, and off-site urgent care facility with a total of 60 EM faculty. The institution also has an emergency US division with a US fellowship and dedicated emergency ultrasonographer. All EM attendings had been trained in ultrasound either during residency or in the practice pathway per ACEP guidelines. After surveying faculty for a needs assessment, nine basic emergency US applications (see Figure 1) were reviewed over the course of a year by US fellowship-trained EM faculty in a simulation center. Each session included 30-minutes of didactics/image review where technique, normal, and abnormal findings were reviewed. The next 30-minutes were used for hands-on practice on normal volunteers. Sessions were optional and scheduled immediately after faculty meetings in order to maximize attendance. An anonymous questionnaire filled out at the end of each session evaluated comfort level performing and supervising the ultrasound application before and after the course using a Likert scale from 1 “not at all confident” to 5 “very confident”. The survey also investigated years of practice, moonlighting, personal use of US for the application over the last year, and supervision of trainees over the last year.

The results were compiled anonymously by a blinded reviewer. Statistical analyses were done using Microsoft Excel (version 16.16.1). Confidence scores were reported using means, (using 95% confidence intervals (CI)), demographic data were reported using medians, (using interquartile ranges (IQR)), and percentages were used for descriptive data. Differences between two groups were compared using the Student’s t-test with p < 0.05 representing a statistically significant difference.

## Results

Thirty-six EM physicians (60%) participated in at least 1 of the 9 sessions (median 3, IQR 2-4). A total of 84 surveys were completed. Faculty who attended had a median of 10 (IQR 7-15) years in practice. Sixty-one percent of the faculty who attended work at both the academic and community sites. Eighty percent of participants had performed the specific US application within the last year and 81% had supervised the application.

For all sessions combined, confidence in performing emergency US applications increased from a mean score of 3.3 to 4.4 on the 5 point-Likert scale (difference 1.1, CI (0.94, 1.29), p < 0.001) and confidence in supervising trainees increased from a mean of 3.4 to 4.5 (difference 1.1, CI (0.88, 1.23), p < 0.001). Prior to the sessions 44% (37/84) rated themselves a 4 or 5 on performing and 50% (42/84) rated themselves a 4 or 5 at supervising; after the sessions those numbers rose to 96% (81/84) (difference 52%, CI (40.83, 62.78), p < 0.001) and 93% (78/84) (difference 43%, CI (31.32, 53.01), p < 0.001). For individual sessions, confidence in both performance and application increased for all applications, see Figure 1.

**Figure 2:**
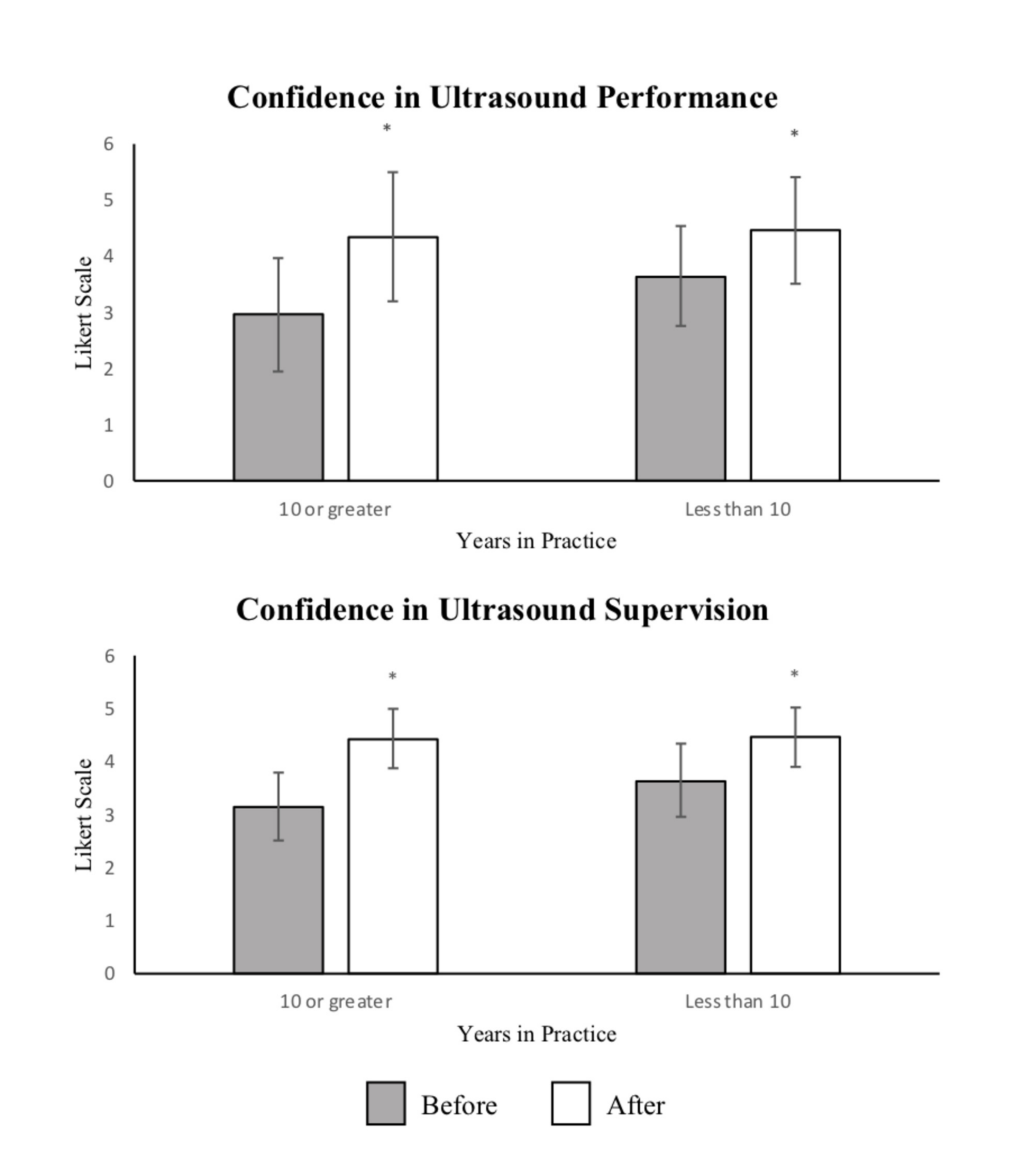
Confidence in Ultrasound Performance and Supervision by Years in Practice Confidence in ultrasound (US) performance and supervision before and after the completion of course by Likert scale (1-5) based on years in emergency medicine practice (10 years or greater versus less than 10 years). Statistically significant difference (p < 0.05) between responses before and after is demonstrated by (*).

Physicians in practice ≥10 years, (55% of respondents), showed an increase in confidence of performing and supervising US by 1.4 (CI (1.11, 1.60), p < 0.001) and 1.3, (CI (1.01, 1.49), p < 0.001), while physicians in practice <10 year, (45% of respondents), increased by 0.8 (CI (0.57, 1.03), p < 0.001) and 0.8 (CI (0.55, 1.05), p < 0.001), respectively, see Figure 2.

## Discussion

This study demonstrated that confidence in both performing and supervising POCUS applications improved in EM physicians after participation in the refresher course. Confidence in performing and supervising was similar both before and after the sessions. This correlation suggests that by improving confidence in performing POCUS, confidence in supervising trainees also increases. This is important because supervising mid-level providers and trainees is a large part of many practicing EM physician’s roles and increasing supervisor confidence may help to improve learner's experience.

Education literature has shown that competency for learned skills, such as ultrasound and other procedures, can rapidly decay after initial training and that a major influence on this decay is the frequency of practice [16]. It has been demonstrated that learned skills for procedures, even those that are rarer, can be maintained more effectively through targeted practice and repetition [17]. Use and frequency of POCUS in the ED across the United States vary widely based on application, practice site, and provider [18]. Our study supports these findings with confidence varying widely, correlating to the frequency the exam is performed but suggests that confidence can be increased rapidly with a targeted refresher.

This study showed improvement in confidence across applications with some variation. Musculoskeletal (MSK) and nerve ultrasound were the applications that the least number of participants had performed or supervised over the last year and were also the applications that the fewest participants felt comfortable with prior to the sessions. These applications also showed the largest improvement after the sessions, suggesting that comfort is lower in applications that are performed less, but that a refresher has the potential to increase confidence to a higher extent. The application with the least change in confidence was intravenous (IV) access. This may be because this is an essential skill that providers are required to perform on a regular basis. In more technically challenging applications that are also commonly used, such as the cardiac and FAST (focused assessment with sonography in trauma) exam, although most participants had performed or supervised this exam recently, there were still many participants who were not confident prior to the session.

The widespread use of POCUS in the ED has been a more recent phenomenon and integration into the EM residency curriculum has occurred within the last decade [3]. Given the differences in the training environment, physicians may have different exposure to POCUS techniques. In this study, physicians in practice for over 10 years had greater increases in their confidence scores than those in practice for less than 10 years. This greater increase in confidence correlates to lower initial confidence in physicians that had been practicing for greater than 10 years. The reported confidence after the teaching sessions then equalizes between these two groups, suggesting that these teaching sessions can bring participants of a variety of prior experience to a similar level of comfort.

With ever-increasing demands of patient care, trainee education, and productivity, efficient learning strategies need to be implemented that balance these competing interests [19]. In this study, the refresher course consisted of short, hour-long sessions spread throughout the year. Given that each session was a stand-alone application, participants were able to benefit from attending any number of sessions. Prior studies that have investigated US education involve single, longer sessions and are often meant for trainees with less experience [11-12]. Given that most practicing EM physicians have already had exposure to POCUS, a short refresher course was thought to be more appropriate and practical. This study demonstrates that short, focused sessions for US with a longitudinal component are effective and this model can be implemented at other institutions for emergency medicine faculty.

Limitations of this study include that it was performed at a single academic center with a small sample size. Participation was optional and the differences between faculty who chose to participate and those that did not, were not analyzed. There was no evaluation of skills or technical improvement with this intervention and there was no long-term follow up into whether the increase in confidence reported translated into measurable clinical outcomes including the increasing number of scans performed or supervised or the quality of future scans. Potential future investigations would be to correlate to US scan numbers, resident or mid-level supervision, and accuracy of scans before and after the intervention.

## Conclusions

A longitudinal US refresher course for EM physicians at a single institution improved self-reported confidence in both performing and supervising trainees in all applications reviewed. Those in practice ≥10 years showed the largest increases in confidence.
